# Implementation of medication alert systems and pharmacist satisfaction: a questionnaire-based study

**DOI:** 10.1186/s40780-026-00611-0

**Published:** 2026-07-17

**Authors:** Takashi Omoto, Manato Inagaki, Junichi Asaka, Kenzo Kudo

**Affiliations:** 1 Department of Pharmacy, Iwate Medical University Hospital, 2-1-1 Idaidouri, Yahaba-cho, Shiwa-gun, Iwate, 028-3695 Japan; 2 Division of Clinical Pharmaceutics and Pharmacy Practice, Department of Clinical Pharmacy, School of Pharmacy, Iwate Medical University, 1-1-1 Idaidouri, Yahaba-cho, Shiwa-gun, Iwate, 028-3694 Japan

**Keywords:** Medication alert systems, Questionnaire-based study, Alert fatigue

## Abstract

**Background:**

As a component of clinical decision support systems, medication alert systems (MAS) play an important role in preventing medication errors. However, their effectiveness is often limited by alert fatigue. Comprehensive data on the implementation and use of specific MAS functions in clinical practice in Japan are limited. Therefore, this study aimed to clarify the implementation of specific MAS functions and identify challenges in real-world use.

**Methods:**

This was a questionnaire-based study conducted among pharmacists at 65 hospitals in Iwate Prefecture, Japan. This questionnaire assessed MAS implementation, functionality, and pharmacists’ satisfaction with MAS, while distinguishing between alerts for physicians (primary alerts) and pharmacists (secondary alerts). Satisfaction was assessed using a 7-point Likert scale. Responses were summarized using medians and interquartile ranges (IQR).

**Results:**

Of the 65 hospitals, 41 responded to the questionnaire (response rate: 63.1%). 85.4% of hospitals implemented a primary alert system, and 80.5% implemented a secondary alert system. Drug–drug contraindication checks were widely implemented for primary and secondary alerts (91.4% and 97.0%, respectively), whereas variations in the implementation of disease contraindication checks and drug allergy checks were observed between primary and secondary alerts (51.4% vs. 33.3%, and 91.4% vs. 36.4%, respectively). The median overall satisfaction score among pharmacists for secondary alerts was 5 (IQR: 3–5). Lower satisfaction scores were observed for disease contraindication (median: 2; IQR: 1.5–4) and drug allergy checks (median: 2.5; IQR: 2–3.5). Excessive alerts, discrepancies between alert content and clinical judgment, and system-related limitations were the most common reasons for dissatisfaction.

**Conclusions:**

Although MAS were widely implemented, variations in alert functions and their use were observed across hospitals and between physicians and pharmacists. Pharmacists commonly reported excessive alerts, discrepancies between alert results and clinical judgment, and system-related limitations as reasons for dissatisfaction. Lower satisfaction was observed for disease contraindication and drug allergy checks, suggesting that these functions require further improvement. These findings highlight the need to optimize alert settings, improve system integration, and enhance the clinical relevance of alerts. Addressing these challenges may help promote the effective use of MAS and improve medication safety in clinical practice.

## Background

Medication errors remain a major concern in healthcare, posing significant risks to patient safety and leading to adverse drug events (ADEs), prolonged hospital stays, and increased healthcare costs [1, 2]. A previous study conducted in Japan identified 1,010 ADEs and 514 medication errors (incidence: 17.0 and 8.7 per 1,000 patient-days, respectively) during the study period [3]. Among these ADEs, 1.6% were fatal, 4.9% were life-threatening, and 33% were serious. Notably, 14% of these ADEs were considered preventable. Medication errors can occur at various stages, including prescribing, dispensing, and administration.

Medication alert systems (MAS) have been introduced as essential clinical decision support systems integrated into electronic health record (EHR) and computerized physician order entry systems to mitigate the risk of medication errors [4–7]. MAS provide real-time alerts on the EHR interface to notify prescribing physicians of potential medication-related risks, such as drug–drug interactions, allergies, and inappropriate dosages. In some hospitals, MAS are integrated with pharmacy department systems, enabling pharmacists to receive similar alerts. Previous surveys of hospitals accredited by third-party accreditation organizations in Japan have reported that most hospitals (99.6%) have implemented MAS [8].

MAS can improve medication safety. A recent pilot randomized controlled trial implementing EHR-based alerts for inappropriate direct oral anticoagulant prescriptions demonstrated high acceptance rates among prescribers [4]. However, alert fatigue poses a significant challenge in MAS implementation [9, 10]. Alert fatigue occurs when an excessive number of irrelevant alerts are generated, leading users to habitually override them. This overriding behavior stems from poor signal-to-noise ratio caused by unserious, irrelevant, or repeatedly displayed alerts [9]. A recent systematic review reported that the alert override rates during order entry ranged from 46.2% to 96.2% [11]. Consequently, clinically important alerts may be overlooked, leading to ADEs [12].

Despite the recognized importance of MAS and the existing challenges, detailed data on how individual MAS functions are implemented, used, and perceived in clinical practice in Japan are lacking. Particularly, the availability and use of specific check items, the practical challenges associated with their operation, and the use of MAS implemented in pharmacy department systems have not been sufficiently investigated. Therefore, this study aimed to clarify the implementation status of specific MAS functions at hospitals in Iwate Prefecture and identify challenges associated with their real-world use.

## Methods

### Study design and participants

This questionnaire-based study targeted 65 hospitals with pharmacists who were members of the Iwate Society of Hospital Pharmacists as of June 1, 2025. Respondents were pharmacists responsible for managing MAS within their hospitals. For facilities without MAS, respondents were pharmacists responsible for drug information management. The questionnaire was mailed on January 30, 2026, with a response deadline of February 27, 2026. Responses were collected via mailed paper questionnaire or through an online survey administered using Google Forms (Google, Mountain View, CA, USA). This study was approved by the ethics committee of Iwate Medical University (MH2025-083) and was conducted in accordance with the Declaration of Helsinki. Informed consent was obtained by including a checkbox for agreement within the questionnaire.

### Questionnaire

**Fig. 1:**
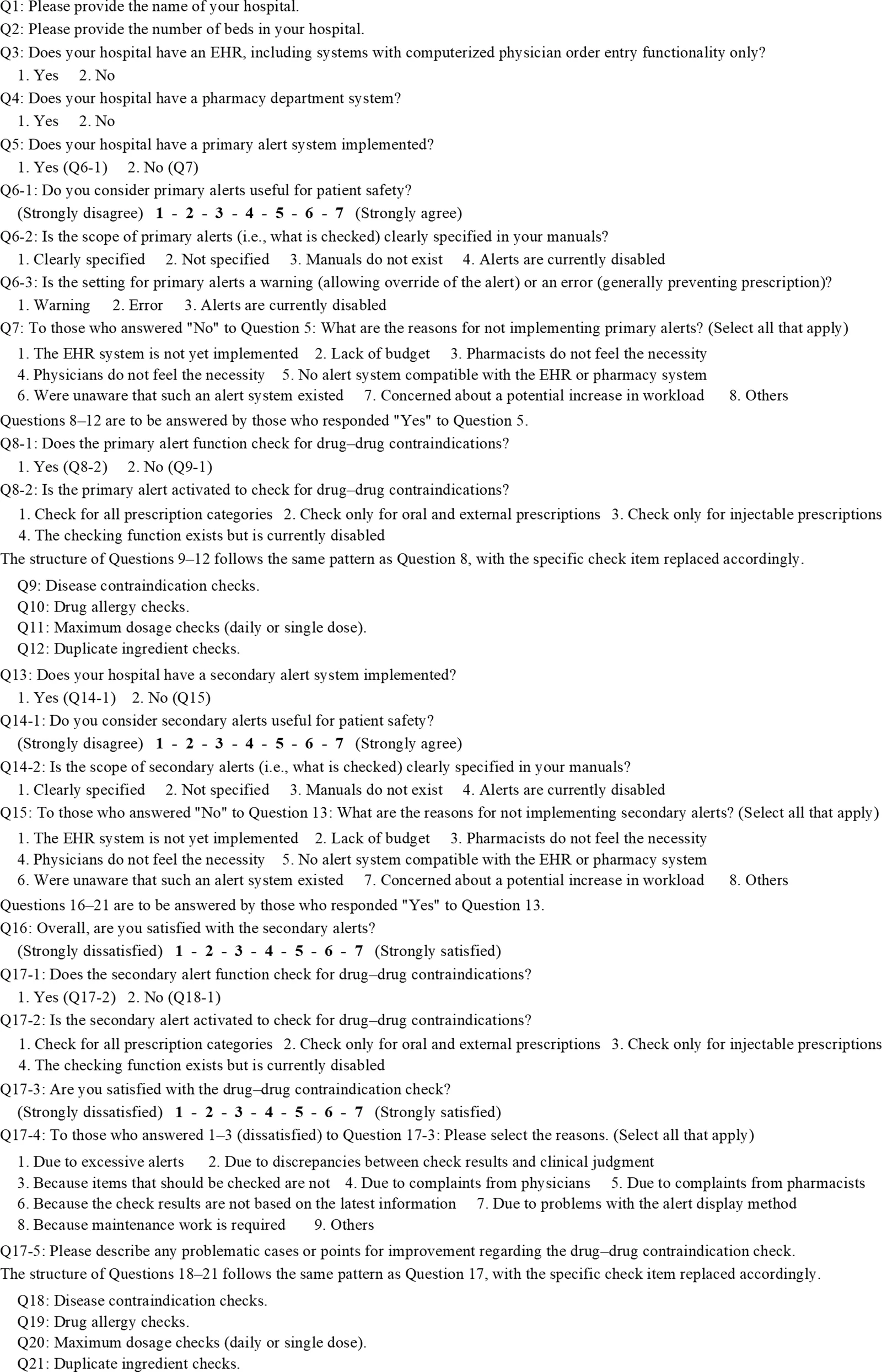
MAS questionnaire

Figure 1 shows the questionnaire items. The questionnaire assessed MAS implementation status and functionality of MAS, as well as pharmacists’ satisfaction with MAS. MAS were defined as system functions that automatically assess prescribed medications for potential issues, such as contraindications and inappropriate dosing, and generate alerts on the EHR or prescription interface when such issues are detected.

Pharmacists’ satisfaction with specific MAS-related items was measured using a 7-point Likert scale ranging from 1 to 7, with 1 indicating strongly dissatisfied, 2 indicating dissatisfied, 3 indicating slightly dissatisfied, 4 indicating neutral, 5 indicating slightly satisfied, 6 indicating satisfied, and 7 indicating strongly satisfied. Responses for each item were summarized using medians and interquartile ranges (IQR).

**Fig. 2:**
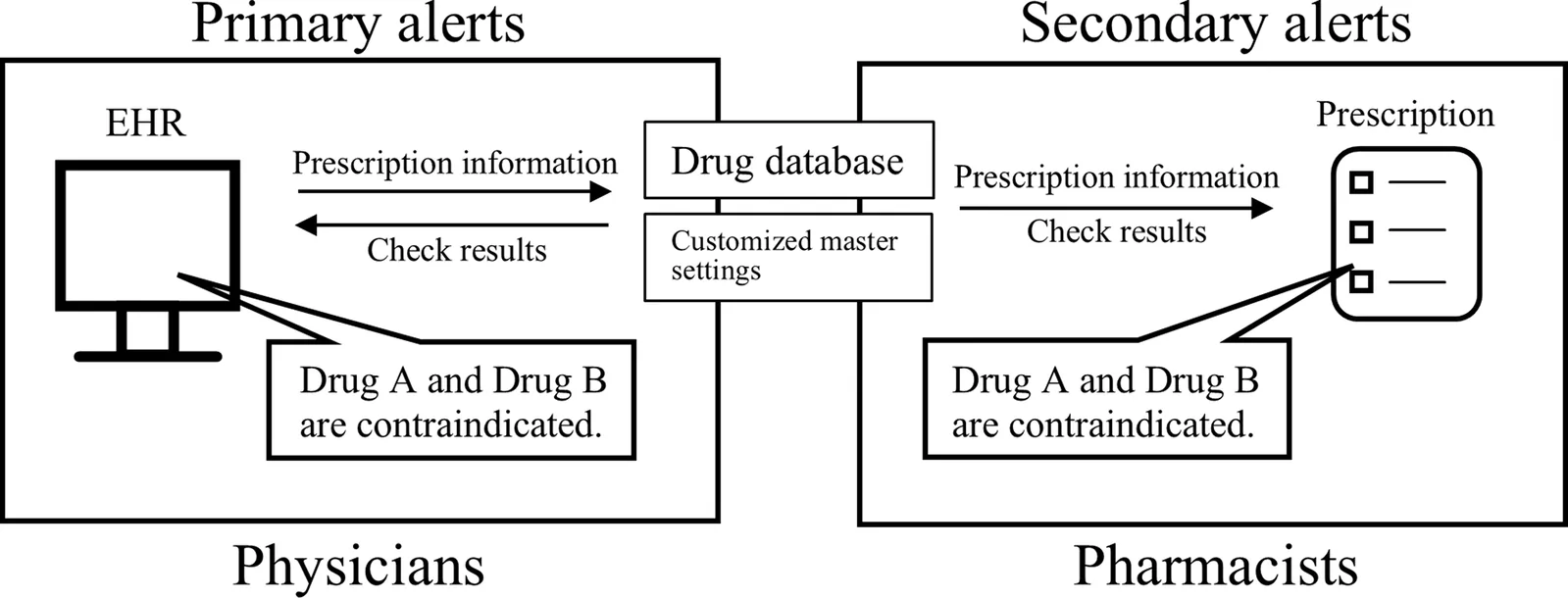
Definition of the primary and secondary alerts

Alerts displayed to physicians were defined as “primary alerts,” whereas those displayed to pharmacists were defined as “secondary alerts” (Fig. 2). Primary alerts were generated from prescription information and checked against the drug database or customized master settings, and the results were displayed on the physician’s EHR. Similarly, secondary alerts were generated from prescription information and checked against the drug database or customized master settings, and the results were displayed to pharmacists through a pharmacy department system or directly on the prescription. An example of such an alert is “Drug A and Drug B are contraindicated.”

## Results

Of the 65 targeted hospitals, 41 responded to the questionnaire (response rate: 63.1%). The median number of beds per hospital was 180 (IQR: 98–249). Regarding EHR implementation (including computerized physician order entry-only systems), 39 (95.1%) hospitals had an EHR. Additionally, 36 (87.8%) hospitals had a pharmacy department system.

Table 1 shows the MAS implementation status. Regarding MAS implementation, 35 (85.4%) hospitals had a primary alert system, and 33 (80.5%) had a secondary alert system. The median perceived usefulness for patient safety was 6 (IQR: 6–7) for primary and secondary alerts. Regarding the specification of alert scope in manuals, 8 (22.9%) hospitals reported that the scope of primary alerts was clearly specified, and 7 (21.2%) reported that the scope of secondary alerts was clearly specified. Regarding the primary alert setting, 33 (94.3%) hospitals used a warning setting.

**Table 1:** Summary of responses regarding MAS implementation

	Primary alerts	Secondary alerts
MAS implementation (Q5, Q13)		
Yes, *n* (**%**)	35 (85.4)	33 (80.5)
Alert usefulness for patient safety (Q6-1, Q14-1)		
Median score (IQR)	6 (6-7)	6 (6-7)
Specification of alert scope in manuals (Q6-2, Q14-2)		
Clearly specified / Not specified Manuals do not exist / Alerts are currently disabled, *n* (**%**)	8 (22.9) / 22 (62.9) 5 (14.3) / 0 (0)	7 (21.2) / 18 (54.5) 8 (24.2) / 0 (0)
Alert setting (Q6-3)		
Warning / Error / Alerts are currently disabled, *n* (**%**)	33 (94.3) / 2 (5.7) / 0 (0)	-
Drug-drug contraindication checks		
Functionality exists (Q8-1, Q17-1)		
Yes, *n* (**%**)	32 (91.4)	32 (97)
Activation status (Q8-2, Q17-2)		
All prescription categories / Only for oral and external Only for injectable / Disabled, *n* (**%**)	28 (87.5) / 2 (6.3) 0 (0) / 2 (6.3)	26 (81.3) / 5 (15.6) 0 (0) / 1 (3.1)
Disease contraindication checks		
Functionality exists (Q9-1, Q18-1)		
Yes, *n* (**%**)	18 (51.4)	11 (33.3)
Activation status (Q9-2, Q18-2)		
All prescription categories / Only for oral and external Only for injectable / Disabled, n (**%**)	12 (66.7) / 0 (0) 0 (0) / 6 (33.3)	6 (54.4) / 0 (0) 0 (0) / 5 (45.5)
Drug allergy checks		
Functionality exists (Q10-1, Q19-1)		
Yes, *n* (**%**)	32 (91.4)	12 (36.4)
Activation status (Q10-2, Q19-2)		
All prescription categories / Only for oral and external Only for injectable / Disabled, *n* (**%**)	28 (87.5) / 0 (0) 0 (0) / 4 (12.5)	6 (50) / 0 (0) 1 (8.3) / 5 (41.7)
Maximum dosage checks		
Functionality exists (Q11-1, Q20-1)		
Yes, *n* (**%**)	26 (74.3)	26 (78.8)
Activation status (Q11-2, Q20-2)		
All prescription categories / Only for oral and external Only for injectable / Disabled, *n* (**%**)	10 (38.5) / 11 (42.3) 1 (3.8) / 4 (15.4)	11 (42.3) / 10 (38.5) 0 (0) / 5 (19.2)
Duplicate ingredient checks		
Functionality exists (Q12-1, Q21-1)		
Yes, *n* (**%**)	21 (60)	27 (81.8)
Activation status (Q12-2, Q21-2)		
All prescription categories / Only for oral and external Only for injectable / Disabled, *n* (**%**)	16 (76.2) / 3 (14.3) 0 (0) / 2 (9.5)	16 (59.3) / 9 (33.3) 0 (0) / 2 (7.4)

The reasons for not implementing primary alert systems (*n* = 6) were “Lack of budget” (*n* = 3), “The EHR system is not yet implemented” (*n* = 2), “No alert system compatible with the EHR or pharmacy system” (*n* = 1), and “Were unaware that such an alert system existed” (*n* = 1). The reasons for not implementing secondary alert systems (*n* = 8) were “Lack of budget” (*n* = 3), “No alert system compatible with the EHR or pharmacy system” (*n* = 2), “The EHR system is not yet implemented” (*n* = 1), and “Were unaware that such an alert system existed” (*n* = 1).

Table 1 also summarizes the functionality and activation status of the primary and secondary alerts. For primary alerts, drug–drug contraindication, disease contraindication, drug allergy, maximum dosage, and duplicate ingredient checks were implemented in 32 (91.4%), 18 (51.4%), 32 (91.4%), 26 (74.3%), and 21 (60%) hospitals, respectively. For secondary alerts, drug–drug contraindication, disease contraindication, drug allergy, maximum dosage, and duplicate ingredient checks were implemented in 32 (97%), 11 (33.3%), 12 (36.4%), 26 (78.8%), and 27 (81.8%) hospitals, respectively.

For primary and secondary alerts, the proportions of hospitals in which drug–drug contraindication, disease contraindication, drug allergy, maximum dosage, and duplicate ingredient checks were activated for all prescription categories were 28 (87.5%) and 26 (81.3%), 12 (66.7%) and 6 (54.5%), 28 (87.5%) and 6 (50.0%), 10 (38.5%) and 11 (42.3%), and 16 (76.2%) and 16 (59.3%), respectively. Disease contraindication checks were disabled in 6 (33.3%) hospitals for primary alerts and 5 (45.5%) for secondary alerts, drug allergy checks were disabled in 5 (41.7%) hospitals for secondary alerts, and maximum dosage checks were activated only for oral and external prescriptions in 11 (42.3%) hospitals for primary alerts and 10 (38.5%) for secondary alerts.

Table 2 shows pharmacists’ satisfaction with secondary alerts. The median overall satisfaction score was 5 (IQR: 3–5). The median satisfaction scores were 5 (IQR: 3–6) for drug–drug contraindication checks, 2 (IQR: 1.5–4) for disease contraindication checks, 2.5 (IQR: 2–3.5) for drug allergy checks, 4 (IQR: 2.25–5) for maximum dosage checks, and 4 (IQR: 3.5–5.5) for duplicate ingredient checks.

**Table 2:** Satisfaction levels with secondary alerts

	Score							Median (IQR)
	1	2	3	4	5	6	7	
Overall, *n* (Q16)	3	2	4	6	10	4	4	5 (3–5)
Drug-drug contraindication checks, *n* (Q17-3)	3	2	5	2	7	9	4	5 (3–6)
Disease contraindication checks, *n* (Q18-3)	3	4	0	2	1	1	0	2 (1.5–4)
Drug allergy checks, *n* (Q19-3)	2	4	3	0	1	2	0	2.5 (2–3.5)
Maximum dosage checks, *n* (Q20-3)	4	3	3	6	5	2	3	4 (2.25–5)
Duplicate ingredient checks, *n* (Q21-3)	3	3	1	7	6	6	1	4 (3.5–5.5)

**Fig. 3:**
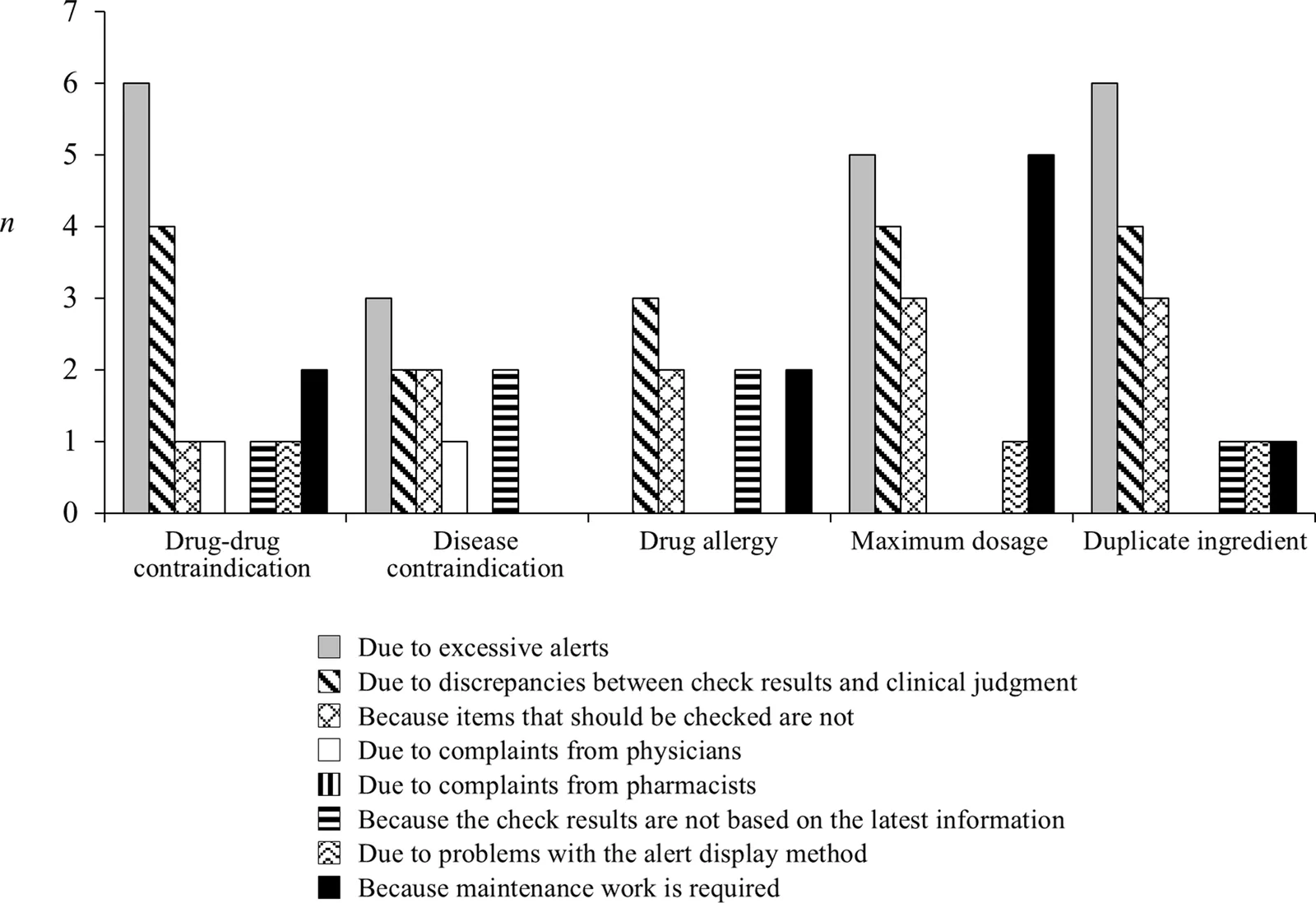
Reasons for pharmacists’ dissatisfaction with secondary alert check items

Figure 3 shows the reasons for dissatisfaction among pharmacists who were dissatisfied with the specific checks. The most frequently reported reasons were “Due to excessive alerts” (*n* = 6) and “Due to discrepancies between check results and clinical judgment” (*n* = 4) for drug–drug contraindication checks, “Due to excessive alerts” (*n* = 3) for disease contraindication checks, “Due to discrepancies between check results and clinical judgment” (*n* = 3) for drug allergy checks, “Due to excessive alerts” (*n* = 5) and “Because maintenance work is required” (*n* = 5) for maximum dosage checks, and “Due to excessive alerts” (*n* = 6) and “Due to discrepancies between check results and clinical judgment” (*n* = 4) for duplicate ingredient checks.

Table 3 shows pharmacists’ reasons for dissatisfaction with each check item. The most common reasons for dissatisfaction were the lack of linkage between systems, the requirement for manual data entry, excessive alert frequency, and discrepancies between check results and clinical judgment.

**Table 3:** Reasons for pharmacists’ dissatisfaction and suggested improvements for each alert check item

	Reasons for dissatisfaction and suggested improvements
Drug-drug contraindication checks(Q17-4, 17 − 5)	・Lack of checking for interactions between prescribed medications and patients’ own medications ・Maintenance burden ・Unclear presentation of check results
Disease contraindication checks (Q18-4, 18 − 5)	・Lack of linkage of diagnosis information between the EHR and pharmacy system, requiring manual registration in the pharmacy system ・Excessive alerts leading to uncertainty in clinical judgment
Drug allergy checks (Q19-4, 19 − 5)	・Lack of linkage of drug allergy information between the EHR and pharmacy system, requiring manual registration in the pharmacy system ・Uncertainty in distinguishing true drug allergies from reported allergies
Maximum dosage checks (Q20-4, 20 − 5)	・High frequency of alerts ・Variation in appropriate dosage depending on the disease
Duplicate ingredient checks (Q21-4, 21 − 5)	・Lack of checking for duplicate therapy between prescribed medications and patients’ own medications ・Duplicate alerts between outpatient and inpatient prescriptions

## Discussion

This study investigated MAS implementation status and challenges in hospitals in Iwate Prefecture, Japan. Furthermore, this study examined MAS in detail by categorizing them into primary and secondary alerts and by individual check items. The results showed that MAS were implemented in more than 80% of hospitals. However, previous studies have reported a significantly higher implementation rate (99.6%) [8]. This discrepancy may be due to differences in the study populations. The previous survey included hospitals accredited by third-party organizations with a focus on patient safety [8], whereas our survey targeted hospitals within a single prefecture. This study population was not restricted to a specific group and included hospitals with various functions, including those without EHR. Therefore, the findings may better reflect real-world clinical practice. These findings suggest that differences in hospital characteristics may contribute to variation in MAS implementation.

The results showed that primary and secondary alerts differed in their functions and utilization. Drug–drug contraindications were implemented and used in primary and secondary alerts. However, disease contraindications were implemented in a limited number of hospitals. Additionally, drug allergy checks were often unavailable, particularly in secondary alerts. These findings indicate that the available check items vary across different MAS and between physicians and pharmacists. Only approximately 20% of hospitals specified the scope of MAS alert items in their institutional manuals. Therefore, at the institutional level, sharing which items are being checked throughout the workflow from prescribing to dispensing is necessary. Previous studies have highlighted the importance of alert management [8]. To the best of our knowledge, this is the first study to demonstrate that the checkable items vary based on the MAS implemented at each hospital and that these items differ between primary and secondary alerts.

Disease contraindication and drug allergy checks were disabled in a substantial proportion of hospitals. This finding highlights a gap between system capability and actual clinical use. These alerts may generate an extensive number of irrelevant or low-value notifications, leading users to disable them. Consistent with this, pharmacists reported lower satisfaction with these functions than with the other check items. These functions rely on patient-specific clinical information, the availability and accuracy of which may affect their clinical relevance. In routine clinical practice, however, maintaining accurate and continuously updated disease information and allergy histories in a format that can be recognized by the system remains challenging. Therefore, the limited implementation and frequent deactivation of these functions may reflect not only insufficient clinical information but also practical difficulties in maintaining such information.

Respondents also identified system-related factors, including lack of integration between the EHR and pharmacy systems, the need for manual data entry, and the inability to check prescribed medications against patients’ own medications, as reasons for dissatisfaction. These findings suggest that both system interoperability and workflow design require further improvement. Future improvements may require enhanced interoperability between information systems and workflow designs that facilitate the continuous recording and updating of patient clinical information. For example, integrating clinical information documented during routine pharmaceutical care, such as medication management records prepared by ward pharmacists, into MAS may improve the availability and accuracy of disease and allergy information.

Furthermore, discrepancies between MAS check results and pharmacists’ clinical judgment were also frequently reported as a reason for dissatisfaction. This finding suggests that improving the quality and clinical relevance of alert functions is important in addition to reducing unnecessary alerts. Therefore, MAS vendors should refine these alert functions to better align alert generation with real-world clinical practice, thereby enhancing their clinical relevance and reducing unnecessary alerts.

Alert fatigue has been recognized as a major issue affecting the usability of MAS [9, 10]. Although alert fatigue was not directly evaluated in the present study, pharmacists frequently perceived excessive alerts as a reason for dissatisfaction with multiple check items. Frequent alerts with low specificity can increase clinicians’ workload and lead to the dismissal of alerts [13, 14]. A previous study showed that 65.3% of drug–drug interaction alerts were overridden by physicians, indicating that a significantly high proportion of alerts are not acted upon [15]. Consequently, even clinically important alerts may be overlooked, potentially increasing the risk of ADEs. Therefore, reducing unnecessary alerts and improving the signal-to-noise ratio are crucial for enhancing the effectiveness of MAS.

This study has some limitations. First, the sample size was relatively small and limited to hospitals in a single prefecture. Therefore, further large-scale studies are needed. Second, although hospital size was collected, the impact of hospital size and system vendors on MAS implementation and usability was not evaluated in the present study. Future studies should investigate the influence of these factors on MAS implementation and user satisfaction. Third, the results were based on self-reported data, which may be subject to reporting bias. Because objective measures of alert fatigue, such as alert frequency, override rates, and acceptance rates, were not collected, the present findings should be interpreted as pharmacists’ perceptions of alert burden rather than direct evidence of alert fatigue. Future studies should incorporate quantitative metrics, such as alert frequency, override rates, and acceptance rates. However, such data are not always readily accessible [8], indicating that system-related limitations should be considered and further explored. Fourth, physicians’ satisfaction with or perspectives on MAS were not assessed. Physicians are key users of MAS, and their input is important for system improvement. Therefore, future studies should investigate physicians’ perspectives. Finally, the extent of institution-specific customization of MAS to improve system quality in each hospital was not evaluated. For example, implementation and configuration of renal function-based dosage alerts, which are important for medication safety and may influence pharmacist satisfaction, were not investigated in the present study. Understanding the status of such customization across institutions can help identify more specific challenges associated with MAS.

## Conclusions

MAS were widely implemented. However, alert functions and their use varied across hospitals and between physicians and pharmacists. Pharmacists commonly reported excessive alerts, discrepancies between alert results and clinical judgment, and system-related limitations as reasons for dissatisfaction. Lower satisfaction was observed for disease contraindication and drug allergy checks, suggesting that these functions require further improvement. These findings underscore the importance of optimizing alert settings, improving system integration, and enhancing the clinical relevance of alerts. Addressing these challenges may enhance the effective use of MAS and improve medication safety in clinical practice.

## Data Availability

The datasets used and/or analyzed during the current study are available from the corresponding author upon reasonable request.

## Abbreviations

ADEs: Adverse drug events
EHR: Electronic health record
IQR: Interquartile range
MAS: Medication alert systems
